# A Cross-Sectional Analysis of the Incidence of Hydatidiform Mole in Colombia

**DOI:** 10.1155/ogi/8899358

**Published:** 2025-02-08

**Authors:** Mario Arturo González Mariño

**Affiliations:** Department of Obstetrics and Gynecology, Faculty of Medicine, Universidad Nacional de Colombia, Bogotá, Colombia

**Keywords:** births, Colombia (MeSH), hydatidiform mole, incidence (MeSH)

## Abstract

**Objective:** To describe the incidence of hydatidiform mole in Colombia.

**Design:** Cross-sectional descriptive study.

**Setting:** Colombia.

**Population or Sample:** The total population at risk of hydatidiform mole (15–49 years old) from 2015 to 2023 was 117.890.729 women.

**Methods:** A search of the national database of the Ministry of Health and Social Protection of Colombia using ICD-10 for hydatidiform mole and ectopic pregnancy was conducted from 2015 to 2023. Incidences were calculated using data from the National Department of Statistics of Colombia.

**Main Outcome Measures:** The incidence of hydatidiform mole was calculated by ratios compared with the live births, the proportion of pregnancies, and the incidence rate in the at-risk population.

**Results:** In the reviewed period, 2247 cases of hydatidiform mole were reported. The proportion of unspecified hydatidiform moles during the evaluation period was 78.59% of the reports, the incidence ratio was one hydatidiform mole for every 2486 live births, the proportion was 37.7 × 10^5^ pregnancies and the cumulative incidence was 1.90 × 10^5^ women of 15–49 years. The age range with the highest number of cases was 20–29 years with 1039 cases.

**Conclusions:** The high proportion of unspecified hydatidiform mole far exceeds the diagnoses of complete and partial hydatidiform mole. The descriptive design of the study does not allow us to determine the causes of these results. Future studies with more complex methodological designs are required to explain these findings.

## 1. Introduction

Gestational trophoblastic disease is a rare group of pathologies associated with pregnancy that exhibit abnormal trophoblastic proliferation; with the development of premalignant and malignant pathologies [1, 2]. Premalignant include complete hydatidiform moles (CHMs) and partial HMs (PHMs), the most common forms of gestational trophoblastic disease (80%) [3].

Molar pregnancies are derived from villous trophoblast composed mainly of cytotrophoblast and syncytiotrophoblast. They are characterized by hydropic edema of the chorionic villi and trophoblast proliferation [1]. The HM can be classified as partial when maternal chromosomes are present or complete when they are absent [4].

A diagnosis of CHM is suspected using ultrasound and quantitative measurement of serum human chorionic gonadotrophin (hCG) with markedly elevated levels [1]. In ultrasonography, the HM appears as a uterus filled with vesicles and absence of a gestational sac. However, these features are not visible early. Large studies have shown that only 40%–60% of moles are detected by ultrasound in clinical practice [5]. Furthermore, 10% of the cases that were reported as moles (complete and partial) on ultrasound were diagnosed as nonmolar hydropic abortions on histological review [6].

The partial mole is usually triploid (69 chromosomes) with two paternal and one maternal contributions to the nuclear genome. Early ultrasound diagnosis of partial HM is more difficult than CHM because it can mimic normal early pregnancy [1].

In some cases, ancillary techniques are needed, including immunostaining for P57^kip2^, which is expressed by the maternal allele and is visible on histology as nuclear staining of cytotrophoblast and villous mesenchyme in the placenta of all pregnancies, except in complete androgenetic mole [3, 7]. Ploidy analysis by in situ hybridization or flow cytometry can distinguish diploid from triploid conceptions, helping to diagnose partial mole, but it cannot distinguish a complete mole from diploid nonmolar miscarriage or molar versus nonmolar triploid, which requires molecular analysis [5].

The incidence of gestational trophoblastic disease varies widely worldwide. The incidence of HM varies between 0.57 and two per 1000 pregnancies according to studies in North America, Australia, New Zealand, and Europe [8]. Brazil reported 4.65 per 1000 pregnancies [9]. In Colombia, previous data were based on hospital cases reporting between one per 155 and one per 680 pregnancies [10].

CHM appears to be caused by abnormal gametogenesis and fertilization, which occur more frequently at the extremes of reproductive age [1]. Compared with women aged 21 to 35, the risk of complete mole has been reported to be 1.9 times higher in women under 21 years of age and 7.5 times higher in those over 40 years [1]. Maternal age is not associated with the risk of partial mole [11].

In addition to the CHM and PHM, the International Statistical Classification of Diseases and Related Health Problems (ICD-10) includes the HM, unspecified, for cases that were HM, but a further classification into partial or complete was not made [12].

This study of HM collected registry data from the Ministry of Health and Social Protection of Colombia and population distribution estimated by the National Administrative Department of Statistics of Colombia (DANE) [13]. The incidence was calculated and compared according to the number of live births, pregnancies, and population at risk from 2015 to 2023.

## 2. Methods

### 2.1. Data Sources

A cross-sectional descriptive study was performed using the national database of the Individual Service Provision Registry data (RIPS) from the Ministry of Health and Social Protection of Colombia. The HM codes (O01.0 classic, O01.1 partial, and O01.9 unspecified) of the ICD-10 from January 1, 2015, to December 31, 2023, were searched. The incidence rates were calculated using the ratio of the number of HMs reported with the number of live births per year and also with the proportion of HM in the number of pregnancies obtained by adding the live births, stillbirths (including miscarriages and abortions), reported by the DANE, and ectopic pregnancies obtained from RIPS. Additionally, the incidence of HM was calculated in women aged 15–49 years from the data of DANE.

DANE's data on live and stillbirths are obtained from certificates completed in physical or digital media by physicians or authorized personnel. The RIPS data are required by the General Social Security Health System for various purposes, including financial purposes. Information for the RIPS is provided by all health sectors (public, private, or mixed) to the corresponding government health offices.

### 2.2. Outcomes

The incidence rates of HMs were calculated using epidemiological ratios, proportions, and rates in the population at risk (15–49 years old) in 10^5^ women.

### 2.3. Statistical Analysis

Statistical analyses were conducted using descriptive statistics and frequency measures.

### 2.4. Ethical Considerations

This is a risk-free research that uses data based on public statistics. Individuals were not evaluated.

## 3. Results

The annual distribution of live births, stillbirths, ectopic pregnancies, and their sum which gives the number of pregnancies per year are shown in Table 1. Also shown in this table are the number of HMs per year in Colombia and the annual population of women. 15–49 years during the years 2015–2023, with which the incidences are calculated. The year with the highest number of HMs was 2019 (366 cases) and the year with the lowest number was 2016 (155 cases).

The incidences of HM in Colombia measured by ratios, proportions, and rates are presented in Table 2, and the distributions of pregnancies, HMs, and their proportions according to age are presented in Table 3.

In the years reviewed (2015–2023), 2247 cases of HM were reported. The proportion of unspecified moles during the evaluation period corresponded to 78.59% of the total reports of HMs, with complete moles accounting for 13.48% and partial moles accounting for 7.92%. The age range with the highest number of cases of HM was 20–29 years (1039 cases). For complete mole, the sum of the number of cases diagnosed in those under 20 years of age and in those over 40 was 73 (24% of the cases diagnosed as complete mole).

## 4. Discussion

### 4.1. Main Findings

Three epidemiological measures were calculated to determine the incidence of HM in Colombia. The ratio was used to relate it to live births and pregnancies. The result of this epidemiology measure is often expressed as the result “to one” [14] as shown in Table 2. However, some studies about the incidence of the HM are referred to 1000 live births, so a simple conversion is required, giving an incidence ratio in the span evaluated of 0.40 per 1000 live births, which is lower than those found in the study by Tham et al. [15] of 1.33 per 1000 live births in the north of England and Wales among the non-Asian population and 2.02 per 1000 live births on average in a Japanese study [16].

The incidence of HM in pregnancy is a better measure than the ratio. However, it is often presented as a ratio when, in fact, it is a proportion. HM is a pathological manifestation of pregnancy; thus, it is included in the number of pregnancies. Although the previous observations and the fact that they are not comparable because of the addition in this study of ectopic pregnancies, a ratio of 0.37 per 1000 pregnancies was lower than the reports shown above [8, 9] and the ratio of 1:607 found in the study by Savage et al. [17].

The incidence rate was calculated according to risk ages, specified as women aged 15–49 years, as defined by the World Health Organization (WHO) in the analysis of reproductive age. In the study review of age distribution, there were only 8 cases between 12 and 15 years. The incidence rate of 1.90 × 10^5^ in Colombia is lower than that reported in 16 regions of Japan, 13.72 × 10^5^ (ages 15–54) [18].

The results presented in Table 1 show the predominance of complete versus partial mole, which may be related to limitations in the available diagnostic resources. With the widespread use of first-trimester ultrasonography (Colombia's guidelines recommend a first ultrasound between 10 and 11 weeks of pregnancy), most products of conception specimens are encountered at much earlier gestational ages when histological features are less well developed [19], making morphologic features not reliable for the diagnosis of partial and complete moles between them and with other nonmolar gestations [20].

In countries with easier access to diagnostic technologies in addition to morphological evaluations, a greater proportion of partial moles are evident with the use of immunohistochemistry and molecular genotyping (short tandem repeat genotyping) [21].

HM, unspecified, comprised the most number of HM diagnosis in this registry. Histological morphological criteria do not always enable a definite diagnosis which is why a systematic policy of rereading slides for all suspicious moles is recommended [22]. In The Netherlands, the incidence rate of unspecified moles gradually decreased mostly after the introduction of p57^kip2^ immunohistochemistry suggesting improved diagnostic analyses [12, 21]. Some cases are more difficult to diagnose, particularly those with CHM in early gestation and those with PHM. Therefore, ancillary techniques including immunostaining for p57, should be used to identify CHM, that lack p57. Additionally, to establish a more accurate diagnosis of these cases, it is recommended to determine the molecular genotype of each suspected case. In some places, ploidy analysis is used to separate diploid conceptions from triploids, but this analysis is not advisable because of its inferior performance in the analysis of the products of conception.

Diagnosis is important for determining the risk of persistent gestational trophoblastic disease and clinical follow-up [23] which is important to detect progression to gestational trophoblastic neoplasia, developed in approximately 15%–20% of patients with CHM and 1%–5% with PHM [24] and whose diagnosis is based on persistent or rising serum hCG [25]. The duration of hCG monitoring varies according to the histological type and regression rate of hCG [1].

Although the highest number of CHM from the RIPS data registry was found in women aged 20–29 years, when the pregnancy's proportion according to age was calculated, the higher proportion was in the 50–54 years, coinciding with other publications [1, 17].

#### 4.1.1. Strengths and Limitations

In Colombia, there is a central registry of births (DANE) and central databases with information about national health events (RIPS) that allow data comparison with international reports. There are national norms regarding the obligation of all healthcare sectors (public, private, or mixed) to report diagnostic information to the corresponding health offices, from where it is finally directed to the Ministry of Health and Social Protection. However, the figures might not be an exact representation of clinical practice because despite mandatory compliance norms and sanctions in case of noncompliance it is passive epidemiological surveillance that may have the usual limitations of this system [26].

### 4.2. Interpretation

Epidemiological studies have reported a wide variation in the incidence of HM [1], which may depend on underreporting, differences in diagnostic or notification criteria, noncentralized databases [8], use of different denominators or epidemiology measures, variations in diagnostic technology [27], and difficulty in differentiating nonmolar abortuses [1].

Although the best measure of incidence would be the calculation of the HM relationship to the number of conceptions, these data cannot be determined. However, using data on live births, stillbirths (miscarriages and abortions), and ectopic pregnancies, the number of pregnancies can be calculated. This measure, which is a proportion, allows greater uniformity in the calculation of the incidence of HM.

HM is considered a premalignant pathology that requires specific follow-up according to the diagnosis. To improve its accuracy, one of the recommended approaches is the use of a combination of p57 immunohistochemistry and DNA genotyping [23].

## 5. Conclusion

The incidence of HM is generally imprecise. The use of additional diagnostic procedures in addition to morphological examinations should enable a more precise diagnosis of this pathology. In cases of suspected HM, molecular genotyping is recommended. The largest proportion of cases in Colombia is reported as unspecified HM. Future studies with more complex methodological designs are required to explain these findings.

## Figures and Tables

**Table 1:** Report of live births, stillbirths, ectopic pregnancies, annual population of women aged 15–49 years, and diagnosis of hydatidiform mole per year in Colombia.

Report/year	2015	2016	2017	2018	2019	2020	2021	2022	2023
Live births	660,999	647,521	656,704	649,115	642,660	629,402	616,914	573,625	510,357
Still births	48,734	48,619	44,488	41,098	37,875	33,327	30,709	28,237	24,678
Ectopics	3881	2313	2618	3846	5549	4602	4297	3841	3698
Pregnancies	713,614	698,453	703,810	694,059	686,084	667,331	651,920	605,703	538,733
15–49 years	12,398,315	12,500,946	12,619,145	12,827,457	13,106,817	13,370,210	13,551,303	13,696,347	13,820,189
CHM	35	23	26	42	55	39	31	32	20
PHM	19	15	12	16	26	32	26	23	9
HMU	207	117	134	217	285	259	235	182	130
Total	261	155	172	275	366	330	292	237	159

*Note:* PHM, incomplete or partial hydatidiform mole.

Abbreviations: CHM, classic hydatidiform mole; HMU, hydatidiform mole unspecified.

**Table 2:** Incidence of hydatidiform mole in Colombia using several epidemiology measures.

Live births		Pregnancies		Population at risk (15–49 years)
Measures	Ratio	Proportion	Ratio	Cumulative incidence
Years	Mole: live births	X10^5^		X10^5^
2015	1:2532	36.57	1:2734	2.10
2016	1:4177	22.19	1:4506	1.23
2017	1:3818	24.43	1:4091	1.36
2018	1:2360	39,62	1.2523	2.14
2019	1:1755	53.34	1:1874	2.79
2020	1:1907	49.45	1: 2022	2.46
2021	1:2112	44.79	1: 2232	2.15
2022	1:2420	39.12	1: 2555	1.73
2023	1:3209	29.51	1:3388	1.15
Total	1:2486	37.70	1:2652	1.90

**Table 3:** Pregnancies, hydatidiform moles, and proportions (X10^5^) according to age.

Years	< 20	20–29	30–39	40–49	50–54	No reported
Pregnancies	1,131,185	3,129,654	1,536,799	155,825	1304	
CHM	23 (2.03)	144 (4.60)	86 (5.59)	32 (20.53)	18 (1380.36)	0
PHM	8 (0.70)	82 (2.62)	54 (3.51)	29 (18.61)	5 (383.43)	0
HMU	108 (9.54)	813 (25.97)	532 (34.61)	219 (140.54)	93 (7131.90)	1
Total	139 (12.28)	1039 (32.71)	672 (43.72)	280 (184.82)	116 (8895.70)	1

## Data Availability

The databases consulted are freely accessible to the general public. However, the RIPS database needs to meet several requirements from the Colombian Ministry of Health to obtain the password.
